# Parameter-Based Prioritization of Time-Resolved LC–HRMS Ion Tracks for Untargeted Reaction Monitoring

**DOI:** 10.3390/ijms27198852

**Published:** 2026-10-04

**Authors:** Igor Ilyasov, Arcady Braun, Vera Olicheva, Valery Goman, Victoria Grikh, Anastasiya Zhevlakova, Irina Selivanova, Vladimir Beloborodov

**Affiliations:** 1 Department of Chemistry, Nelyubin Institute of Pharmacy, Sechenov First Moscow State Medical University, Trubetskaya Street 8/2, 119048 Moscow, Russia; olicheva_v_v@student.sechenov.ru (V.O.); goman_v_f@student.sechenov.ru (V.G.); zhevlakova_a_k@staff.sechenov.ru (A.Z.); selivanova_i_a@staff.sechenov.ru (I.S.); vlbe@list.ru (V.B.); 2 Department of Chemistry, Lomonosov Moscow State University, 27/1 Lomonosovsky Avenue, 119991 Moscow, Russia; avbraun@yandex.ru; 3 Lomonosov Institute of Fine Chemical Technologies, MIREA—Russian Technological University, 119571 Moscow, Russia; 4 Department of Analytical, Physical and Colloidal Chemistry, Nelyubin Institute of Pharmacy, Sechenov First Moscow State Medical University, Trubetskaya Street 8/2, 119048 Moscow, Russia; grikh_v_v_1@staff.sechenov.ru

**Keywords:** untargeted analysis, high-resolution mass spectrometry, LC–HRMS, reaction monitoring, time-resolved analysis, ion prioritization

## Abstract

Herein, an automated, parameter-based workflow is introduced for processing time-resolved high-resolution mass spectrometry (HRMS) data. Its utility is demonstrated using the reaction between the ABTS^•+^ radical cation and the polyphenolic antioxidant naringenin. Data were collected using HRMS coupled to high-performance liquid chromatography (HPLC) in negative ion mode. The algorithm compares sequential time-resolved HRMS measurements to generate kinetic curves and calculate parameters for every detected species. Based on these curves and parameters, we can tentatively classify each species as a reagent, intermediate, or product. The algorithm was implemented as Java-based software. As a result, extensive data arrays of 1.3 million *m*/*z*-intensity pairs per experimental series were processed automatically. The workflow generated manageable candidate sets in each replicate series. Cross-replicate matching using a 5 ppm mass tolerance retained 29 ion tracks consistently detected in all three reaction runs for subsequent analysis. These ions can then be examined manually to identify compound structures. Overall, the approach provides a screening tool for untargeted reaction monitoring and supports subsequent structural annotation in complex chemical systems.

## 1. Introduction

Tracking changes in chemical composition within a complex system comprising multiple compounds and dynamic processes is a major analytical challenge. Researchers across disciplines, including metabolomics and pharmaceutical stability studies, often need to capture such changes. High-resolution mass spectrometry (HRMS) is well-suited for this task, as it provides broad coverage of detectable ion signals. The conventional approach to mining such datasets is straightforward: researchers monitor the starting compounds, predict likely intermediates and products, locate them within the HRMS data, and track their concentration trends—while disregarding the vast majority of other ion signals. Conventional manual peak-tracking is routinely employed for HRMS coupled to chromatographic separation. However, this approach suffers from two major drawbacks: it only captures well-resolved, high-intensity signals, and its reliability depends heavily on the researcher’s expertise. Given the size and complexity of the data, the risk of oversight is substantial, necessitating more efficient data processing strategies. The more comprehensive alternative is to conduct untargeted monitoring, which requires laborious processing and dedicated computational workflows to reduce complex LC–MS datasets to a manageable set of features for subsequent interpretation [1,2,3].

Several well-established computational platforms exist for processing liquid chromatography–high-resolution mass spectrometry data, each designed with distinct analytical objectives. XCMS, an R programming library, specializes in statistical comparative analysis, identifying features with significant intensity differences (*p*-values, fold-change) between user-defined sample groups [4]. For time-course experiments, time points are treated as categorical labels, and the output is optimized for hypothesis testing between conditions, not for continuous kinetic modeling. MZmine offers a modular workflow environment for building custom pipelines for peak detection and alignment. While MZmine is exceptionally flexible, kinetic trend analysis requires advanced custom scripting after alignment, as the software lacks dedicated modules for this purpose [5,6]. MS-DIAL is a comprehensive desktop application for universal metabolomics that generates a high-quality aligned feature table from raw data, but the resulting master table is an exhaustive list of all detected compounds that still requires substantial manual curation and external analysis to identify features with systematic kinetic trends—especially for HRMS datasets [7,8]. Compound Discoverer similarly provides an integrated environment for feature detection, alignment, annotation, and statistical analysis, but its primary purpose is comprehensive compound-level data processing rather than parameter-based prioritization of continuous ion trajectories [9].

While these tools are indispensable for data preprocessing, alignment, and differential comparison, there remains a need for automated and readily interpretable prioritization of features according to their behavior across continuous time-series measurements. The analysis of longitudinal untargeted datasets presents an additional challenge, since relationships between consecutive time points may not be adequately represented by conventional pattern-recognition approaches [10].

The present workflow is therefore not intended to replace established LC–MS platforms such as XCMS, MZmine, MS-DIAL, or Compound Discoverer. Rather, it addresses a subsequent and more specific task: rapid prioritization of ion trajectories according to their behavior across an ordered sequence of measurements. Instead of ranking features primarily by between-group statistical significance, annotation confidence, or abundance, the proposed approach constructs time-resolved ion tracks and evaluates them using simple trajectory descriptors, including intensity, segment-wise correlation, MinMax ratio, and Early Change. This makes the method particularly suited to reaction-monitoring experiments in which the analytical objective is to reduce a large feature set to a small number of ions exhibiting chemically meaningful temporal behavior. In this sense, the workflow is complementary to conventional LC–MS preprocessing and feature-analysis software rather than an alternative to it.

A further practical distinction is that the method is implemented as a lightweight standalone Java application designed for direct inspection and filtering of sequential HRMS ion tracks without requiring an R environment, custom scripting, or construction of a broader metabolomics workflow. The emphasis is therefore on transparent, parameter-based reduction of time-resolved datasets rather than comprehensive peak annotation or multivariate metabolomic analysis.

Accordingly, we developed a data processing algorithm that utilizes consecutive HRMS spectra for in situ monitoring of chemical reaction dynamics and applied it to the reaction between 2,2’-azino-bis(3-ethylbenzothiazoline-6-sulfonic acid) radical cation (ABTS^•+^) and naringenin as a proof of concept. The HRMS data from this reaction were organized and filtered to yield a manageable set of ions for subsequent manual interpretation and structural assignment, while comprehensive MS^n^-based structural characterization remained outside the scope of this study.

One of the most frequently used methods for estimating total antioxidant capacity relies on the inhibition of ABTS^•+^ radical cations by an antioxidant [11,12,13]. The ABTS^•+^ radical cation is relatively stable and can be readily generated and monitored spectrophotometrically at 730 nm, which has contributed to the widespread use of this antioxidant-capacity assay. Nevertheless, the underlying chemistry is complex and remains poorly understood, leading to difficult-to-explain stoichiometries and ongoing criticism [12,14,15]. The combination of a large dataset comprising approximately 1.3 million *m*/*z*–intensity pairs and unclear chemical processes made this model an illustrative example suitable for testing and refining the data-processing algorithm. Analyzing such a data array by conventional methods using standard office software packages becomes nearly infeasible. We therefore needed to develop an untargeted analysis algorithm that allows precise tracking of reaction dynamics, capturing not only known compounds but also transient or newly formed species in time-resolved experiments. This paper investigates the use of an automated data-processing approach to prioritize ion tracks exhibiting time-dependent intensity changes for subsequent investigation of potential reactants, products, and transient species.

## 2. Results and Discussion

**Figure 1:**
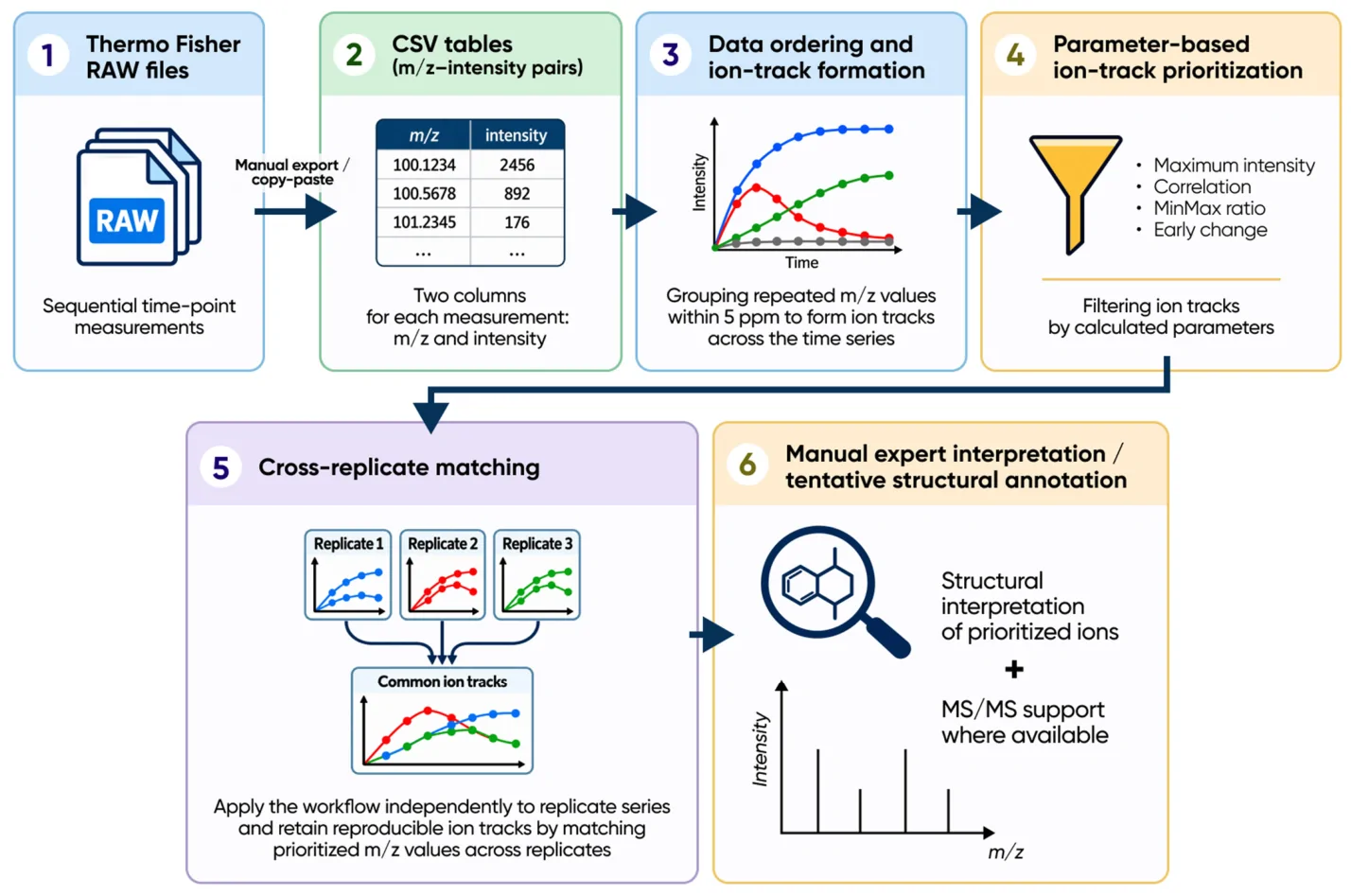
Overview of the complete workflow for processing time-resolved HPLC–ESI–HRMS data. Sequential Thermo Fisher RAW files are manually converted into CSV-formatted *m*/*z*–intensity tables and processed to construct time-resolved ion tracks using a defined mass tolerance. The resulting ion tracks are prioritized according to maximum intensity, correlation, MinMax ratio, and Early Change, followed by cross-replicate matching to retain reproducible signals. The final set of prioritized ion tracks is subjected to manual expert interpretation and tentative structural annotation, with MS/MS evidence used where available.

We developed and implemented a parameter-based workflow for processing time-resolved HPLC–ESI–HRMS datasets and prioritizing ion tracks that exhibit potentially relevant temporal behavior. The complete workflow is summarized in Figure 1. To demonstrate and optimize the workflow, we tested it on the inhibition of ABTS^•+^ radical cations by naringenin. The primary objective was to reduce the full dataset to a manageable number of candidate ions for subsequent structural and mechanistic investigation.

Three replicate reaction time series were acquired under identical experimental conditions. In each series, HRMS *m*/*z*–intensity pairs were collected over a 12.5-h reaction period, with HPLC–ESI–HRMS chromatograms recorded every 30 min following reaction initiation. Each experimental series therefore comprised 26 sequential measurements, forming a continuous dataset that reflected the evolution of the reaction mixture throughout the studied timeframe. Each series contained approximately 1.3 million *m*/*z*–intensity pairs, corresponding to approximately 3.9 million *m*/*z*–intensity pairs across the three replicate experiments.

### 2.1. Algorithm Design and Workflow 

**Figure 2:**
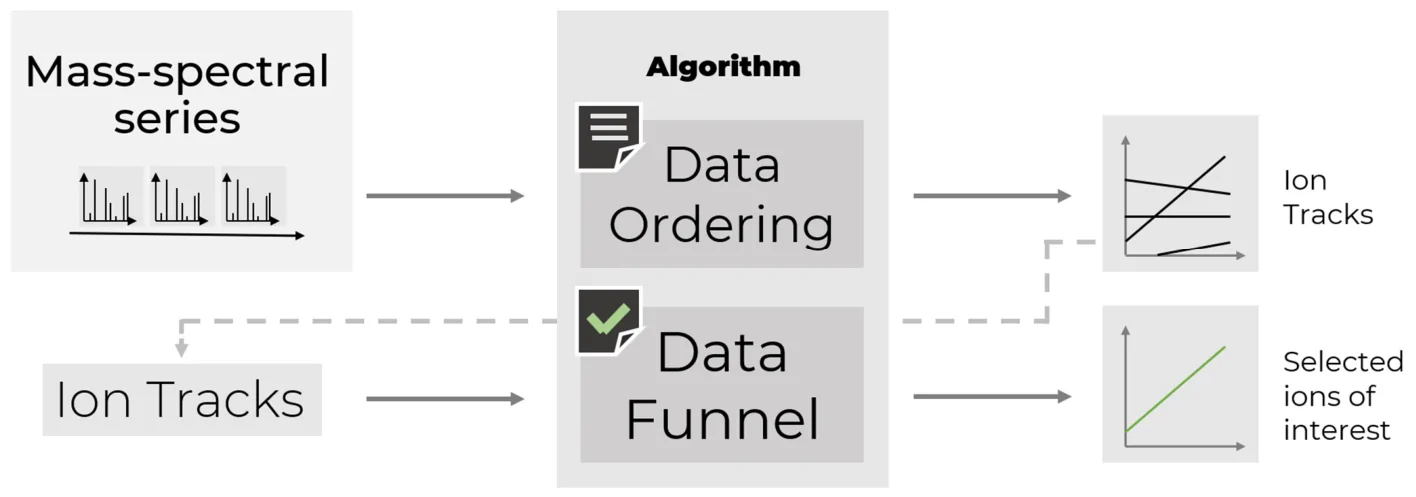
Schematic of mass-spectral series processing through the algorithm’s two independent parts: data ordering, which assembles the data into ion tracks, and the data funnel, which selects tracks meeting specific criteria.

The algorithm comprises two sequential steps. The first step orders the raw *m*/*z*–intensity data across the time series, grouping them into ion tracks, i.e., a set of kinetic profiles that describe the evolution of intensity over time for each detected *m*/*z*. The second step applies a data funnel to these tracks, filtering out irrelevant ions and retaining only the most relevant candidates for further analysis (see Figure 2).

According to the proposed workflow, the data must first be exported from the instrument software and manually organized to obtain a suitable file containing *m*/*z* and intensity data from all measurements in chronological order. The file is then imported into the program for automated processing. First, the algorithm finds and matches unique *m*/*z* values across the series using a configurable mass tolerance (5 ppm in the present study) to compensate for HRMS measurement error. Next, it assigns corresponding intensities and links them to time points for kinetic curve analysis.

**Figure 3:**
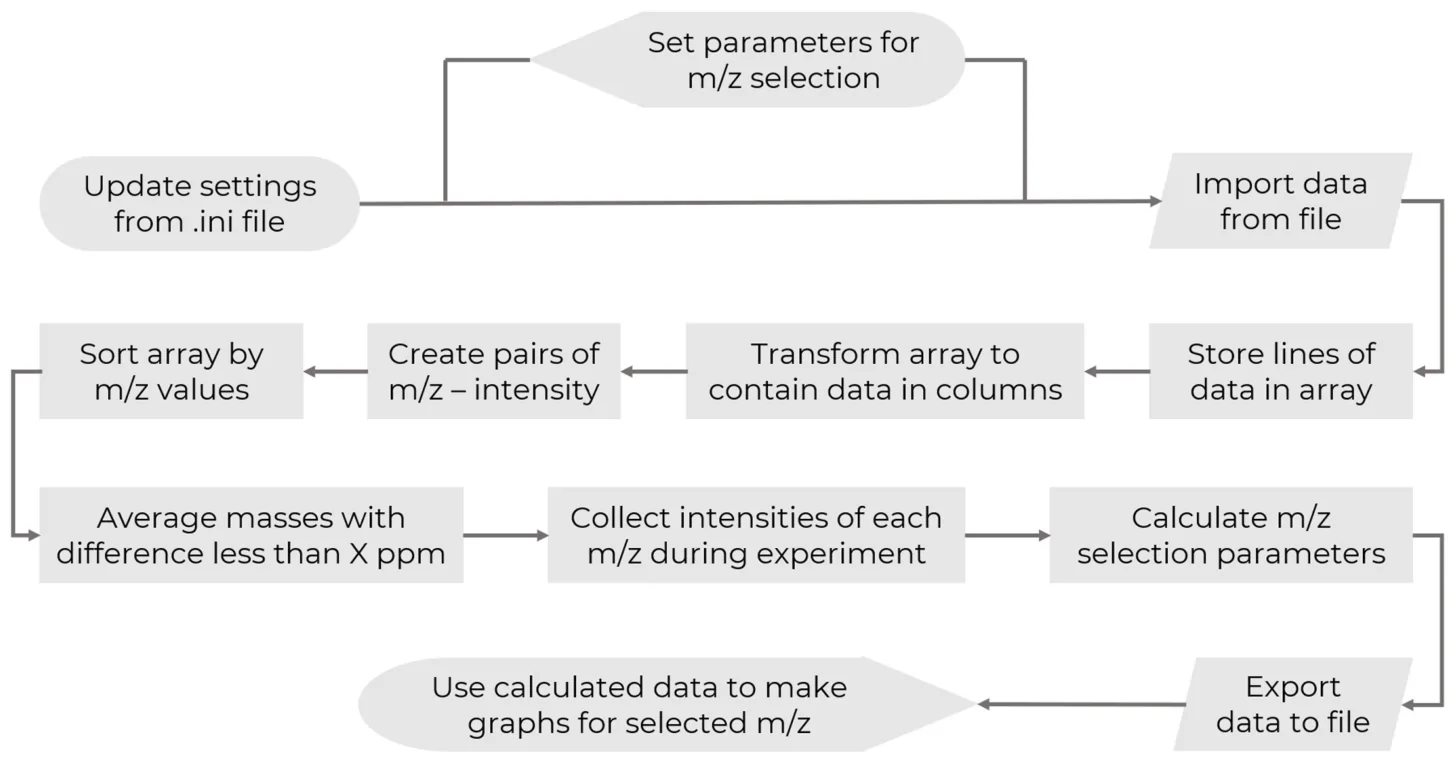
Data ordering algorithm (12 steps).

The data ordering algorithm comprises the 12 steps shown in Figure 3.

### 2.2. Implementation

Initially implemented as an Excel-VBA macro, the algorithm required more than 30 min to process 10,000 *m*/*z*–intensity pairs in the test dataset. To achieve faster calculations, reduced resource demands, and streamlined GUI development, we transitioned to a Java-based implementation. The Java implementation substantially accelerated data ordering, with ion-track construction requiring less than 30 s under the reported test conditions. Notably, neither the CPU nor RAM was utilized at full capacity during the Excel-VBA execution, suggesting the primary bottleneck was MS Excel itself, rather than the PC’s hardware specifications.

The Java application, source code, compiled executable, user documentation, and example input datasets are publicly available at https://github.com/Lefroel/HRMS_time_series_analyser.

Processing the negative ion mode HPLC–ESI–HRMS scans of the naringenin/ABTS^•+^ reaction with the algorithm reduced the dataset from 1.3 million *m*/*z* values in the unprocessed series to 120 thousand unique *m*/*z* values with corresponding ion tracks in the processed series. Nevertheless, the resulting dataset remained too large for practical manual interpretation. Consequently, further processing was needed.

### 2.3. Development of the Data Funnel

The development of the data-funnel workflow is demonstrated below using the first replicate HPLC–ESI–HRMS time series as a representative dataset. To balance the feasibility of manual review against comprehensive data coverage, we aimed to reduce the dataset to ~60 ions. We therefore introduced several parameter-based filters (described below) to group and prioritize the obtained *m*/*z*–intensity tracks.

*Grouping by maximum intensity throughout the track*. The first grouping parameter we implemented was intensity, as it is the simplest. We assign a ranking to each detected *m*/*z* according to the maximum intensity observed across the series. Specifically, the group rank is determined by the order of magnitude, as illustrated by the following example: the “10^5^” rank is assigned to ions whose observed intensity falls within the range from 1 × 10^5^ to 9.(9) × 10^5^.

For the present dataset, the higher-intensity groups provided a convenient starting point because they contained fewer tracks and generally showed more reproducible signals. However, lower-intensity tracks were not discarded *a priori*, since transient products or species with limited ionization efficiency may exhibit pronounced temporal changes despite low absolute abundance. Intensity was therefore used as a prioritization parameter rather than as an irreversible pre-filter.

Although an intensity threshold could substantially reduce the dataset at an earlier stage, we intentionally retained the complete exported signal set during ion-track construction. The purpose of the workflow is not solely to prioritize the most abundant ions, but also to preserve low-intensity species showing pronounced temporal changes, which may correspond to transient intermediates or products with low abundance or limited ionization efficiency. Lower-intensity groups can still be analyzed through targeted approaches, although their much larger size makes it harder to focus on specific *m*/*z* values.

*Finding the steadiest tracks.* The correlation coefficient is a key metric for ranking ions by how consistently their intensity changes over time. In reaction monitoring, we expect to observe a decrease for reagents, an increase for products, and a bell-shaped curve for intermediates. These kinetic trajectories are often fitted with trend lines–commonly linear for reagents and products, and polynomial for intermediates. In practice, however, perfectly linear trends across an entire series are uncommon.

To address this, each ion track is divided into shorter time intervals where linear behavior is more likely. This approach emulates the manual procedure of inspecting individual kinetic curves but avoids the prohibitive effort of evaluating >100,000 tracks one by one. By focusing on shorter intervals, stronger linear trends can be captured with higher correlation coefficients. We use the absolute value of the Pearson correlation coefficient so that both increasing and decreasing trends are scored by proximity to 1. Ions are classified by their best segment-wise correlation: group A, |r| ≥ 0.99; group B, 0.90 ≤ |r| < 0.99; group C, 0.70 ≤ |r| < 0.90; group D, |r| < 0.70.

**Figure 4:**
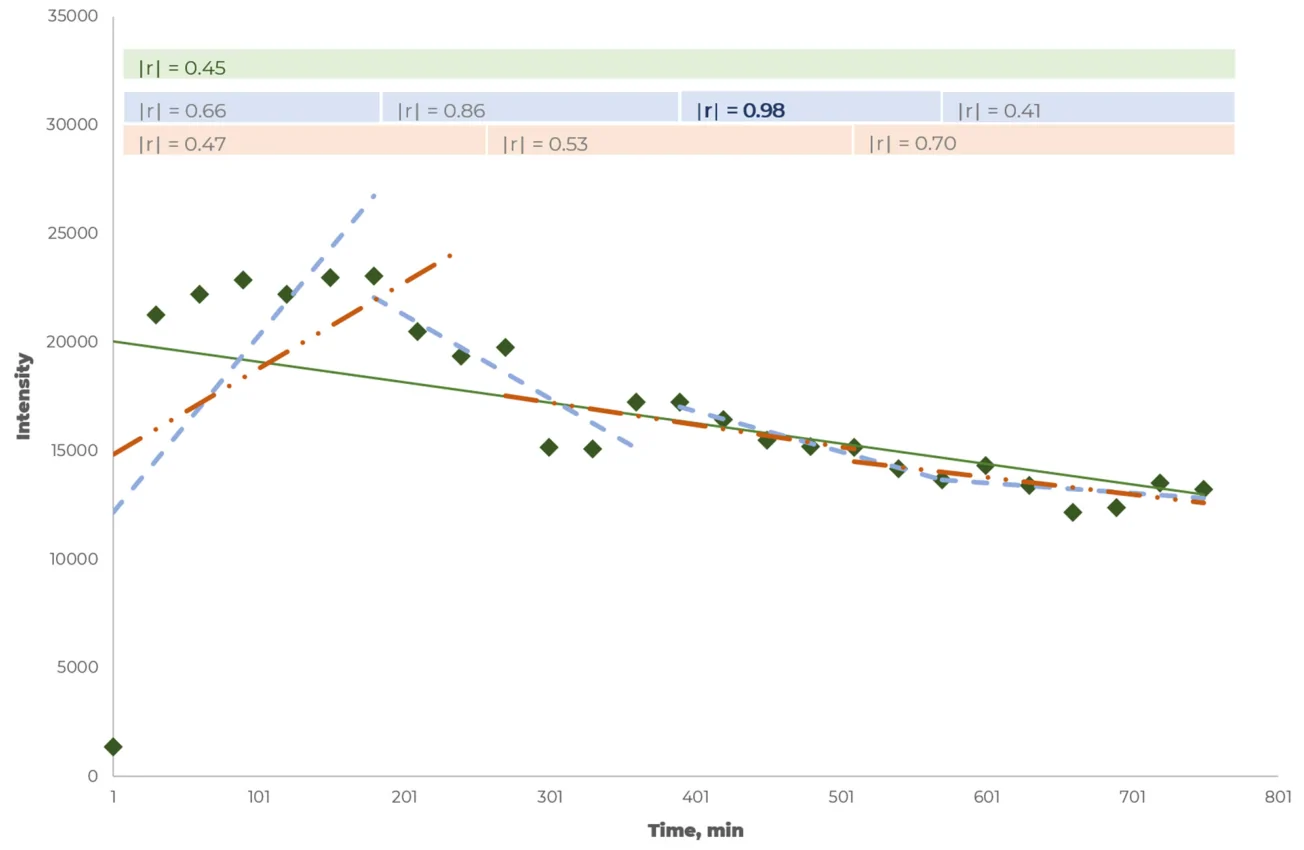
Dividing the timeline for correlation analysis allows us to better capture changes in ion tracks. Green diamond-shaped points represent the ion track at *m*/*z* 540.0534 *in the negative* ion mode HPLC–ESI–HRMS series for naringenin/ABTS^•+^. The solid green line is the linear correlation curve for the whole track; curves for quarters are light blue, and curves for thirds are orange. The boxes show the correlation coefficients for the respective parts of the timeline. The box for the third quarter has the highest correlation coefficient and is therefore highlighted in bold.

To improve trend capture, the timeline is segmented into overlapping windows (e.g., halves, thirds, and quarters). Overlap ensures that trends spanning window boundaries are still detected. To obtain reliable correlation estimates, each segment should include at least six intensity–time pairs. For each ion, the largest |r| among all segments is used to represent its trend (Figure 4).

Initially, we planned to use only the first two parameters, but it soon became apparent that too many ions still exhibited high intensity along with a correlation coefficient approaching 1. However, many of these ions did not demonstrate any substantial changes in intensity. In essence, some of the trend lines were nearly parallel to the *x*-axis (see Figure S1). Such signals may arise from matrix ions, background contaminants, or persistent laboratory-derived compounds that remain constant across scans. This observation motivated a third parameter to remove largely static tracks and highlight ions with meaningful dynamics.

*Filtering out static ions.* To quantify the practical importance of an intensity trend, we implemented a metric based on the magnitude of the relative intensity change. If the difference between minimum and maximum intensities is sufficient, indicating perceptible changes, the parameter value approaches 1. This approach facilitates the identification of ions with substantial changes in relative abundance. The calculations are performed using the following equation:

(1) $$\mathrm{MinMax}\;\mathrm{ratio}\;=\;1-\;\frac{Minimum\;intensity}{Maximum\;intensity},$$

where Minimum intensity is the lowest intensity observed for a given ion track and Maximum intensity is the highest. Based on the calculated MinMax ratio, ion tracks were divided into four groups: group A, MinMax ratio ≥ 0.75; group B, 0.50 ≤ MinMax ratio < 0.75; group C, 0.25 ≤ MinMax ratio < 0.50; group D, MinMax ratio < 0.25.

Applying the intensity, correlation, and MinMax ratio metrics captured most of the relevant ions; however, one important curve pattern was still missing. Many ions exhibited rapid intensity changes during the first three to four time points, which is shorter than the minimum segment size used for correlation analysis. As a result, such tracks were missed. This led us to introduce an additional parameter designed specifically to capture early-stage dynamics–“early change”.

*Capturing early-stage processes.* The correlation metric is well suited to long-term trends but may fail to detect ions that change drastically over short periods. We therefore developed an “early change” metric to identify ions with substantial changes within the first four time points. These early points are particularly informative because the system may not yet have reached equilibrium and fast processes can dominate. Early change is defined as the magnitude of change over the first four points relative to the maximum intensity of the full track. Values near 1 indicate rapid early growth or decay. This metric helps identify compounds that emerge quickly from low/zero abundance or species that rapidly disappear and therefore show low overall correlation. It is also useful for filtering large, low-intensity groups that remain unmanageable after applying the other parameters.

(2) $$\mathrm{Early}\;\mathrm{change}=\frac{\left|1st\;time\;point\;intensity\;-\;N\;time\;point\;intensity\right|}{Maximum\;intensity},$$

where N is the 2nd, 3rd, or 4th time point, chosen to maximize the early change value. The same group thresholds as those defined for the MinMax ratio were applied to the Early Change parameter.

**Figure 5:**
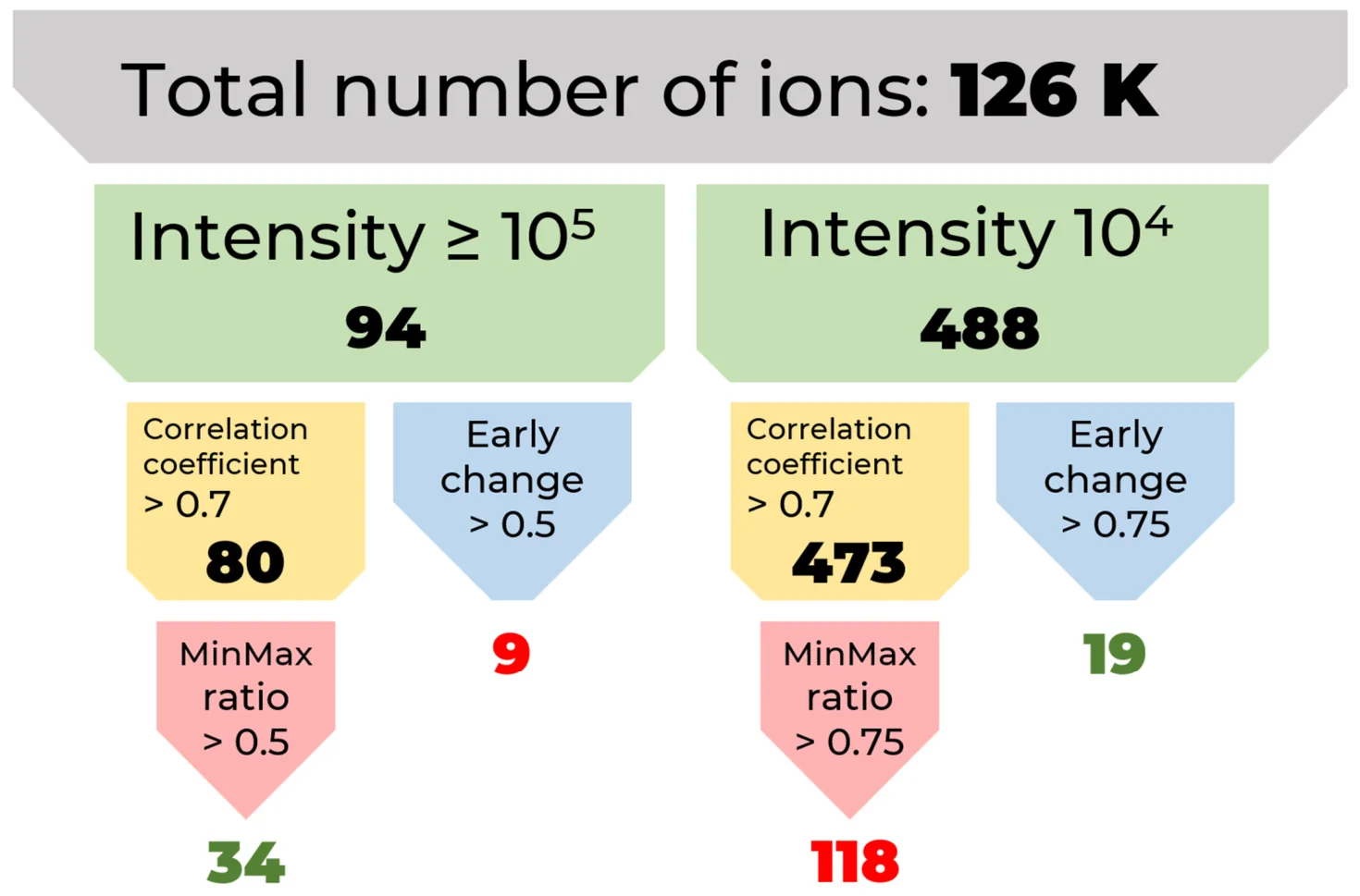
The number of ions retained at each data-funnel filtering stage in the first replicate HPLC–ESI–HRMS series of the naringenin/ABTS^•+^ reaction in negative ion mode. Green highlights the *m*/*z* groups retained for follow-up analysis.

After applying the intensity, correlation, MinMax, and early-change parameters to the ion tracks, the distributions shown in Table 1 and Table 2 and Figure 5 were obtained. Approximately 100 ions had a maximum intensity above 10^5^, which was sufficient for further processing. For the present dataset, we empirically selected |r| = 0.70 as a practical lower threshold for the correlation coefficient (corresponding to the “A”–“C” groups), balancing stable trend selection with the quantitative reproducibility of the Orbitrap analyzer. Eighty ions were selected and filtered based on their MinMax ratio values. The “A” and “B” groups were selected empirically as a practical compromise, because these ions exhibited changes exceeding 50% while yielding a candidate set of manageable size. This analysis strategy resulted in the exclusion of uninformative ion tracks, leaving 34 ion tracks for manual processing. After applying the early change filter for groups “A” and “B” to the 100 ions selected in the initial step, it initially seemed that this parameter did not provide much new information. None of the nine ions were unique. However, we proceeded with the processing of the 10^4^ intensity group, which consists of nearly 500 tracks. Applying the same correlation and ratio filters did not yield a manageable number of ions, and stricter ranges offered little improvement, as the list still contained more than 60 ions. Conversely, processing this set with the early change filter for group “A” proved productive, resulting in the selection of 19 new ions. These threshold values were chosen empirically for this dataset and were not subjected to a robustness analysis.

The prioritization procedure selected 53 tracks from approximately 126,000 in the first replicate, with *m*/*z* values ranging from 112.9555 to 785.0533. Figure 5 summarizes the filtering stages applied to this experimental series.

After the data-funnel workflow had been established using the first replicate, the same processing procedure and parameter thresholds were independently applied to the two additional replicate reaction series. This resulted in 61 and 69 prioritized ion tracks in the second and third replicates, respectively. The prioritized ion lists from all three replicates were then cross-matched by *m*/*z* using a mass tolerance of 5 ppm. Only ions detected in all three replicate series were retained for further interpretation, resulting in a final set of 29 reproducible ion tracks.

**Figure 6:**
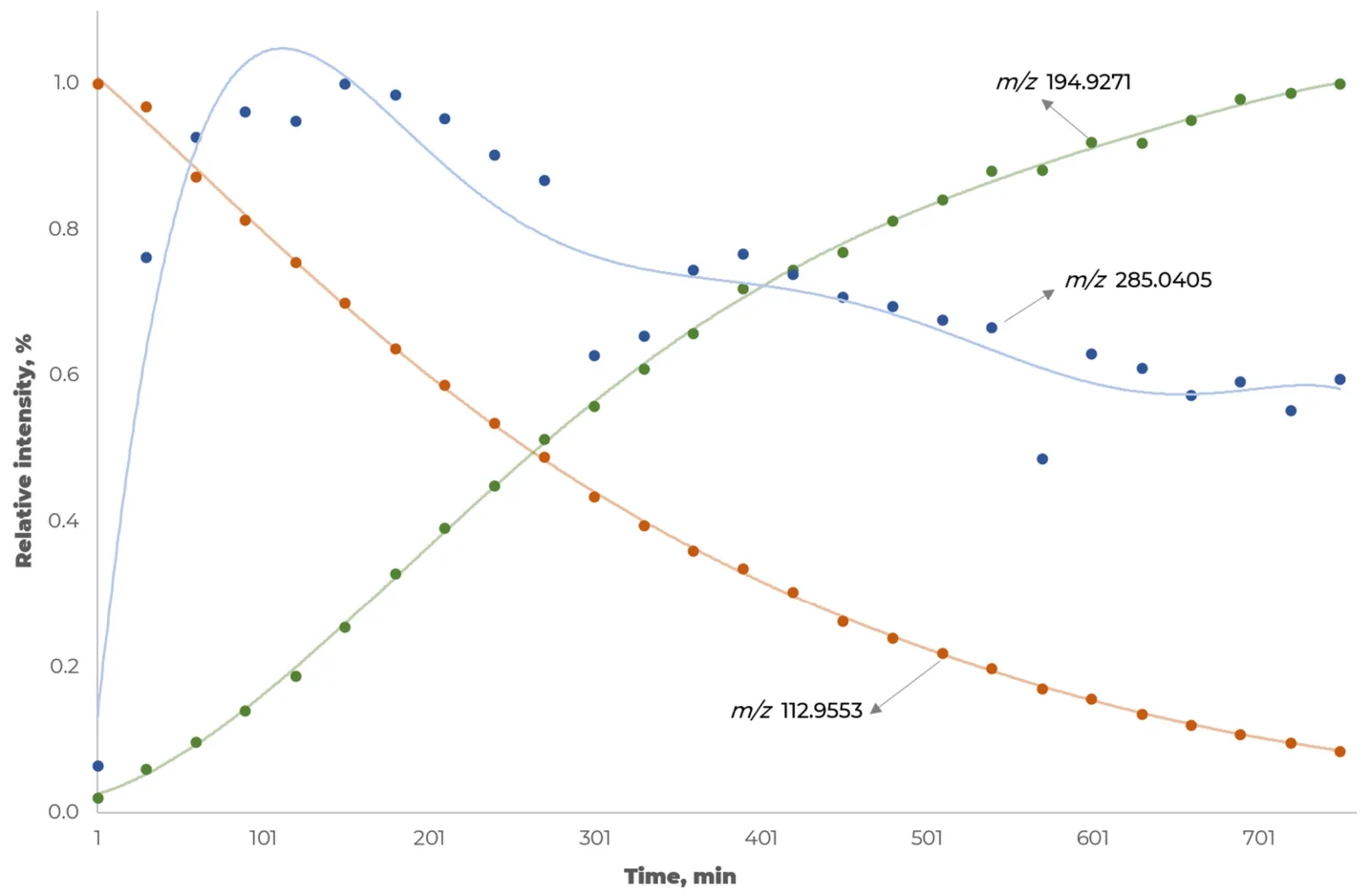
Three main types of track patterns observed after extensive manual analysis of the negative ion mode HPLC–ESI–HRMS dataset for naringenin/ABTS^•+^. The dots in the graph represent the measured relative intensity values at specific experimental time points for the corresponding ions: *m*/*z* 112.9555 shows a declining trend, *m*/*z* 194.9272 a rising trend, and *m*/*z* 285.0406 a bell-shaped trend. The solid curves represent fifth-order polynomial fits used solely as visual guides to facilitate recognition of the overall intensity shifts.

**Figure 7:**
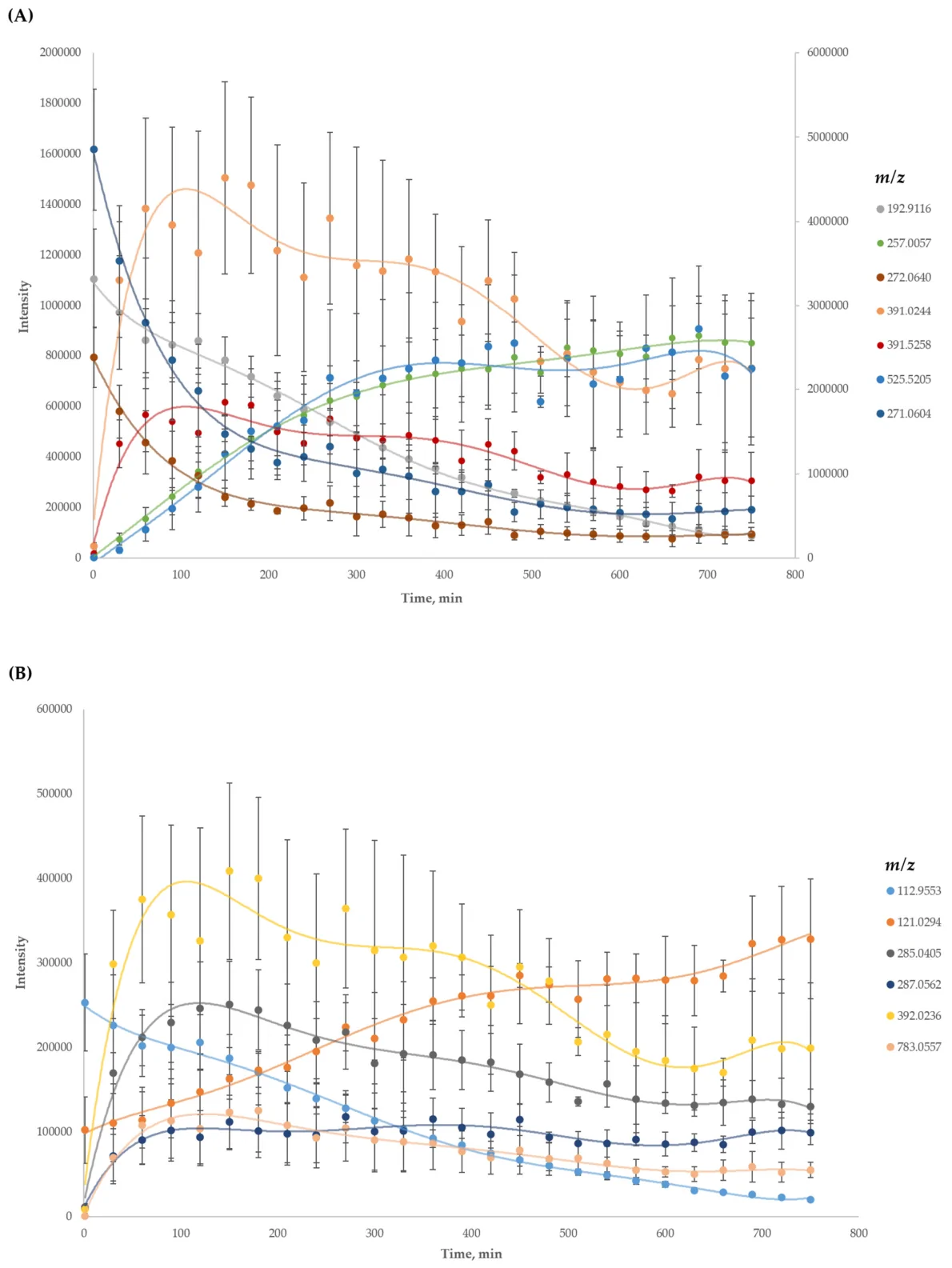

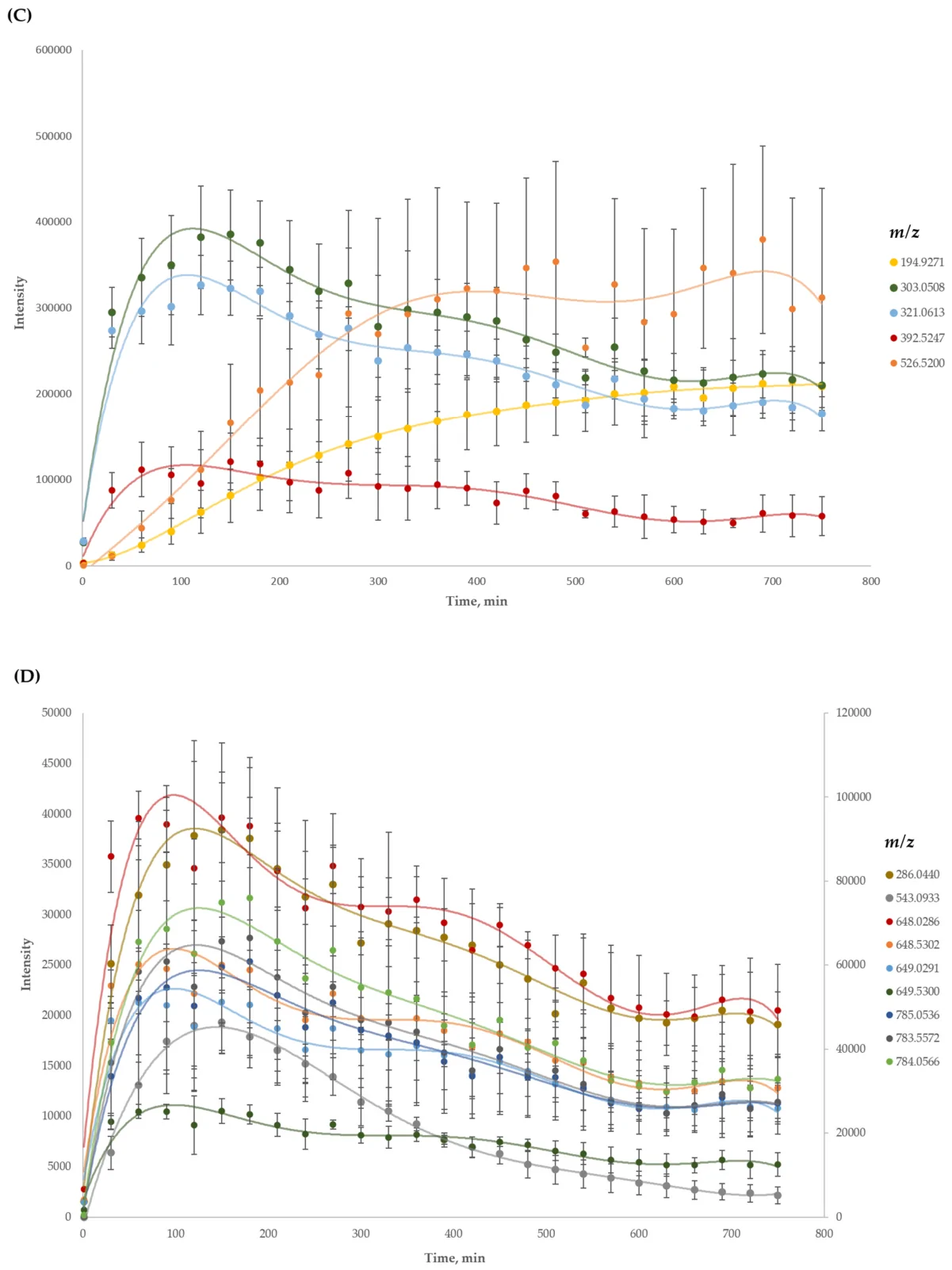
Selected ion tracks in the negative ion mode HPLC–ESI–HRMS series for the naringenin/ABTS^•+^ experiment. The dots in the graph represent mean ± SD across three runs at specific experimental time points for the corresponding *m*/*z* values. The solid curves represent fifth-order polynomial fits used solely as visual guides to facilitate recognition of the overall intensity shifts. Ions with *m*/*z* 271.0604 in panel A and *m*/*z* 784.0566 in panel D were plotted using secondary axes.

*Temporal patterns.* During manual inspection of the selected tracks, we noted that changes in intensity followed specific patterns. Therefore, an attempt was made to categorize these tracks according to the nature of the alterations they exhibited. Preliminary manual inspection indicated declining intensity in 4 ions, which is consistent with the initial compounds that underwent consumption throughout the experiment. Maximum intensity values were consistent with our initial expectations. Ions with higher intensity values primarily exhibited a declining pattern as the reagents are present in the reaction mixture in greater amounts. A rising pattern was observed for 6 ions that accumulated over time. These ion tracks were tentatively interpreted as potential reaction products. Certain ions accumulated and then declined, resulting in a bell-shaped pattern. This was the most prevalent pattern, with 17 ions demonstrating it. We assume that these ions were intermediates of the reaction, which were being formed and consumed concurrently. Ions with lower intensity predominantly followed bell-shaped or rising patterns, as they represented the intermediates or products of the reaction that emerged starting from zero concentration. After examining all the tracks, we identified three major patterns: declining, rising, and bell-shaped (see Figure 6). A graphical representation of selected ions reveals different track patterns (see Table 3 and Figure 7). Two ions did not demonstrate a clear pattern, yet they successfully passed all the filters (see Figure S2). Consequently, they were labeled as “no pattern.” Figure 7 shows the absolute intensities of the ion peaks and how they change over the course of the series. The measurement times for the series match the injection times for the reaction mixture, since the reaction in the sampled volume stops when the chromatographic separation starts. Retention time was not considered in the present workflow, as it was not a primary parameter of the analysis. Chromatographic separation itself was used mainly to reduce matrix effects prior to mass-spectrometric detection. Nevertheless, once ions of interest have been prioritized, their chromatographic behavior can be inspected manually, substantially reducing the amount of chromatographic data requiring expert evaluation. The chromatographic behavior of different ions can thus be similar or different, allowing ions with similar chemical properties to be detected.

The complete workflow, including the replicate-consistency filter, reduced the final set to 29 reproducible ion tracks detected in all three experimental series. In other applications, the strictness and combination of the data-funnel parameters can be adjusted depending on the analytical objective and dataset characteristics. Early change can be applied independently to identify rapid intensity changes that may not be captured by the other parameters.

### 2.4. Tentative Annotation of Representative Prioritized Ions

To demonstrate that the prioritization workflow retains chemically relevant signals, representative ion tracks were subjected to preliminary structural annotation. Table 4 summarizes selected ions corresponding to the starting compound and putative reaction products/intermediates. The prioritized signals included the deprotonated naringenin ion at *m*/*z* 271.0605, an oxidized derivative assigned to a B-ring catechol at *m*/*z* 287.0563, the corresponding B-ring o-quinone at *m*/*z* 285.0406 and its water adduct at *m*/*z* 303.0509, as well as signals attributed to a persulfate ion at *m*/*z* 112.9553 and a half-ABTS moiety at *m*/*z* 257.0057. These assignments demonstrate that the parameter-based workflow retained ions associated with chemically meaningful transformations occurring in the studied reaction. They are provided to demonstrate the analytical relevance of the prioritized ion set rather than to establish a comprehensive reaction mechanism.

Comprehensive structural characterization of the oxidation products, including detailed MS/MS interpretation and reconstruction of the naringenin oxidation pathways, was beyond the scope of the present study, which focuses on the development and evaluation of the ion-prioritization workflow.

### 2.5. Sensitivity to Filtering Thresholds

The correlation and MinMax thresholds employed in the present workflow were selected empirically as practical prioritization criteria rather than as universally validated decision boundaries. To assess the dependence of feature retention on these choices, both thresholds were varied around the values used in the primary analysis while all other processing parameters were held constant (see Table 5). For each tested cutoff, the percentage of retained ion tracks and the retention of the representative chemically annotated ion tracks listed in Table 4 were recorded. Retention of the annotated examples was used as a practical check of whether increasingly stringent thresholds excluded chemically interpretable reaction-related features.

Increasing the stringency of either parameter progressively reduced the proportion of retained ion tracks, whereas more permissive thresholds resulted in broader feature retention. Importantly, all six representative annotated reaction-related ion tracks remained selected throughout the tested range, except at the strictest correlation cutoff of 0.95, where four of the six annotated examples were retained. This indicates that retention of the principal chemically interpretable features was not strongly dependent on a single exact threshold value within the tested range.

It should be noted that, for the purpose of this sensitivity analysis, correlation and MinMax were evaluated as standalone filters. In the full workflow, these parameters are intended to operate in combination with the other prioritization criteria. Consequently, the relatively high proportions of retained ion tracks observed here should not be interpreted as the final degree of dataset reduction achieved by the complete filtering sequence.

Formal chemical false-positive and false-negative rates could not be calculated for the experimental dataset because the identities and reaction relevance of all detected ion tracks are not independently known. In an untargeted reaction-monitoring experiment, treating all unassigned ion tracks as negatives would incorrectly assume that unidentified species are chemically irrelevant. Accordingly, the present analysis evaluates threshold robustness and feature-set stability rather than diagnostic sensitivity and specificity.

### 2.6. Limitations

This study has several methodological limitations. The prioritization step uses only *m*/*z* and temporal signal intensity. Chromatographic peak picking and retention-time alignment are not performed by the current algorithm; the imported data contain *m*/*z* and intensity values, whereas chromatographic information is evaluated manually only after ion-track prioritization. MS/MS evidence, adduct relationships and isotope patterns are not included in the ranking criteria. Consequently, distinct chromatographic species with identical or very similar *m*/*z* values may be conflated, while adducts, isotope signals, and in-source fragments originating from the same parent compound may be retained as separate ion tracks. At the current stage, these relationships require manual expert evaluation. The intensity, correlation, MinMax ratio, and early-change thresholds were selected empirically for the present dataset and should not be interpreted as universally validated cutoffs. Sensitivity to variation in the correlation and MinMax thresholds was evaluated in the present study; however, robustness across independent datasets and chemical systems remains to be established. In addition, the workflow was demonstrated using a single ABTS/naringenin reaction, one HPLC–ESI–HRMS configuration, and negative ion mode. The detected ion set therefore represents only the fraction of the reaction mixture observable under these conditions, and any inferred chemical network should be regarded as partial. Transferability to other chromatographic methods, instruments, ionization modes, reaction systems, and matrices remains to be established.

A direct head-to-head comparison with chromatographic peak-picking, PCA-based, or established untargeted LC–MS workflows was not performed in the present study; consequently, the current results should not be interpreted as demonstrating superior feature recovery relative to those approaches.

The current workflow is also only partially automated because raw-data export and chronological organization are performed manually before software processing. Future evaluation should include automated data import, benchmarking against established LC–HRMS workflows, and validation on independent chemical systems.

## 3. Materials and Methods

### 3.1. Materials

Naringenin was obtained from Acros Organics (Geel, Belgium). ABTS [2,2′-azino-bis(3-ethylbenzothiazoline-6-sulfonic acid) diammonium salt], ammonium bicarbonate, and potassium persulfate (dipotassium peroxydisulfate, PP) were obtained from Sigma-Aldrich (Burlington, MA, USA). HPLC-grade LiChrosolv^®^ ethanol and acetonitrile and puriss. p.a. formic acid (FA) were obtained from Merck (Darmstadt, Germany). Deionized water (18 MΩ·cm) was obtained from LLC Smoly (Moscow, Russia).

All solutions were freshly prepared on the day of use. Antioxidant stock solutions were prepared in ethanol. ABTS was dissolved in 0.01 M ammonium bicarbonate solution (pH 7.4), and PP stock solutions were prepared in deionized water. The solvents did not produce detectable interference under the assay conditions. A 0.1% FA solution was prepared in water and deaerated ultrasonically.

### 3.2. Methods

#### 3.2.1. Software Implementation 

The software was implemented in Java (JDK 22) using IntelliJ IDEA. The graphical user interface was built with Swing and JFreeChart. The application does not directly process the proprietary Thermo Fisher RAW files. Instead, the *m*/*z*–intensity data were manually copied from each RAW file into CSV-formatted tables, which served as the input data for the application. Each measurement was represented by two columns containing the *m*/*z* and corresponding intensity values. In the reported test, ion-track construction required less than 30 s. Testing was performed on a Windows 11 Pro system with an Intel Core i5-12500H processor and 16 GB of RAM, with the Java application executed using default JVM settings.

#### 3.2.2. Algorithm Testing

*ABTS^•+^ scavenging by antioxidants.* The experiment based on ABTS^•+^ scavenging by antioxidants was performed as described in our previous study using the lag-time approach [16,17]. Briefly, an antioxidant sample was added to an ABTS-containing solution prepared in ammonium bicarbonate solution, as described previously. The reaction was initiated by addition of the PP solution, and the mixture was shaken thoroughly for 15 s. Reaction progress was monitored by HPLC–ESI–HRMS.

To reduce variability associated with sample preparation, antioxidant concentrations were selected to produce a lag time of at least 3 min.

*HPLC-ESI-HRMS study of naringenin oxidation.* The analytical system comprised a Dionex Ultimate 3000 RSLC liquid chromatograph equipped with an autosampler and coupled to an Orbitrap Fusion Lumos high-resolution tandem mass spectrometer (Thermo Fisher Scientific, Waltham, MA, USA) with an electrospray ionization (ESI) source. Data were acquired and processed using Xcalibur software (Thermo Fisher Scientific, Waltham, MA, USA).

Under the experimental conditions, the dead time between addition of the radical initiator potassium persulfate and the first mass-spectrometric measurement, including 15 s of shaking and transfer to the autosampler, was approximately 1 min.

After addition of potassium persulfate, the reaction mixture was placed in the thermostated autosampler carousel at 24 ± 1 °C. The aqueous reaction mixture contained 1.3 mM ABTS, 0.4 mM PP, 220 µM naringenin, and ammonium bicarbonate. Aliquots were injected automatically every 30 min and analyzed under the conditions listed in Table 6 and Table 7 until the end of the experiment. Each replicate series comprised 26 measurements acquired at 30 min intervals.

#### 3.2.3. Statistics

The complete naringenin/ABTS^•+^ experiment was performed in three replicate runs under identical experimental conditions. Each replicate series was processed independently using the same parameter settings. The resulting prioritized ion lists were cross-matched using a mass tolerance of 5 ppm, and only ion tracks detected in all three replicates were retained for subsequent interpretation. The intensity values shown in ion-dynamics plots represent the mean ± standard deviation across the three replicate runs.

The absolute value of Pearson correlation coefficient (|r|) was used to describe the regression fit. Fifth-order polynomial regression was applied solely as a visual aid to facilitate recognition of the overall intensity shifts. The fitted curves were not intended to represent a kinetic model or to support quantitative estimation. Original intensity values at individual time points were retained and displayed as data points.

## 4. Conclusions

This approach streamlines the analysis of complex reaction mixtures by transforming a large dataset into a manageable set of candidate ion tracks for subsequent structural investigation. Consequently, it extends the scope beyond targeted analysis, supporting untargeted reaction monitoring and the discovery of candidate reaction-related species for subsequent structural investigation.

*Perspectives*. Future development will therefore include automated pattern recognition and integration of additional analytical dimensions, including isotope/adduct relationships, retention time, and recognition of in-source fragment relationships. Automating raw-data import would also improve reproducibility and scalability. Application to complex matrices and other analytical platforms should be evaluated using independent benchmark datasets.

We envision this tool being applied to a wide range of time-resolved processes, from organic synthesis and catalysis to drug metabolism and biomarker discovery.

## 5. Patents

The software was registered as a computer program with the Federal Service for Intellectual Property of the Russian Federation (Rospatent); Certificate No. 2024664394 was issued on 20 June 2024.

## Figures and Tables

**Table 1:** Ions grouped by order of magnitude of maximum intensity.

Order of Magnitude	10^7^	10^6^	10^5^	10^4^	10^3^	Less
Number of ions	2	15	77	488	2127	123,315

**Table 2:** Number of ions in each group when a single parameter is applied.

Parameter	Group			
	A	B	C	D
Correlation coefficient	703	5324	14,993	105,004
MinMax ratio	122,346	2162	1303	213
Early change	26,344	7505	9979	82,196

**Table 3:** Ions grouped by pattern in the negative ion mode HPLC–ESI–HRMS experiment for naringenin/ABTS^•+^. The reported *m*/*z* values represent the mean values across the three replicate reaction runs. Corresponding ions were matched between replicates using a mass tolerance of 5 ppm.

Pattern	Decline	Rising	Bell Curve		No Pattern
***m*** **/*z***	112.9553 192.9116 271.0604 272.0640	121.0294 194.9271 257.0057 287.0562 525.5205 526.5200	285.0405 286.0440 303.0508 321.0613 391.0244 391.5258 392.0236 392.5247 543.0933	648.0286 648.5302 649.0291 649.5300 783.0557 783.5572 784.0566 785.0536	257.4968 511.9959

**Table 4:** Tentative structural assignments of the representative ion tracks.

*m/z*	Pattern	Ion Formula	Proposed Assignment
112.9553	Decline	HSO_5_^−^	Persulfate ion
257.0057	Rising	C_9_H_9_N_2_O_3_S_2^−^_	Half-ABTS moiety
271.0605	Decline	C_15_H_11_O_5^−^_	Naringenin
285.0406	Bell curve	C_15_H_9_O_6^−^_	Naringenin B-ring o-quinone
287.0563	Rising	C_15_H_11_O_6^−^_	Naringenin B-ring catechol derivative
303.0509	Bell curve	C_15_H_11_O_7^−^_	Naringenin B-ring o-quinone water adduct

**Table 5:** Sensitivity of ion-track retention to correlation and MinMax filtering thresholds. The standard deviation is calculated across three experimental runs.

Correlation Cutoff	Tracks Retained	Annotated Examples Retained	MinMax Cutoff	Tracks Retained	Annotated Examples Retained
0.5	86.6 ± 0.4%	6/6	0.5	99.5 ± 0.2%	6/6
0.6	77.1 ± 3.9%	6/6	0.6	99.1 ± 0.3%	6/6
0.7	68.0 ± 1.8%	6/6	0.7	98.6 ± 0.3%	6/6
0.8	63.2 ± 2.7%	6/6	0.8	97.9 ± 0.4%	6/6
0.85	61.4 ± 2.9%	6/6	0.9	97.0 ± 0.5%	6/6
0.9	59.8 ± 2.8%	6/6	0.99	95.3 ± 0.3%	6/6
0.95	58.5 ± 2.5%	4/6			

**Table 6:** Chromatographic conditions.

Parameter	Value
Column	Acclaim™ 120 C18 (250 mm × 2.1 mm, 3 µm)
Column temperature, °C	24
Mobile phase A	0.1% FA in H_2_O
Mobile phase B	Acetonitrile
Flow rate, mL/min	0.280
Gradient: time (min) → %B	0 → 5; 2 → 5; 15 → 95; 20 → 95; 20.1 → 5; 28 → 5
Injection volume, µL	5

**Table 7:** Mass-spectrometric conditions.

Parameter	Value
Ionization	H-ESI (negative ion mode)
Spray voltage, kV	2.5
Ion transfer tube temperature, °C	325
Vaporizer temperature, °C	350
Sheath gas, arb. units	50
Aux gas, arb. units	10
Sweep gas, arb. units	1
Scan range, *m*/*z*	100–1000
Resolution	60,000
Maximum injection time, ms	30

## Data Availability

The experimental raw data and processed data supporting the findings of this study are provided in the Supplementary Materials. The source code and compiled version of the Java application developed in this study, together with user documentation and a sample input dataset, are publicly available in the GitHub repository: https://github.com/Lefroel/HRMS_time_series_analyser.

## Supplementary Materials

The following supporting information can be downloaded at https://www.mdpi.com/article/10.3390/ijms27198852/s1.
