# Supplementary material for: Parameter-Based Prioritization of Time-Resolved LC–HRMS Ion Tracks for Untargeted Reaction Monitoring
Source: Int J Mol Sci. 2026 Oct 4;27(19):8852. doi: 10.3390/ijms27198852 (PMC13664802; doi:10.3390/ijms27198852)
Supplement: Supplementary file 1 [file ijms-27-08852-s001.zip › Supplementary.pdf]

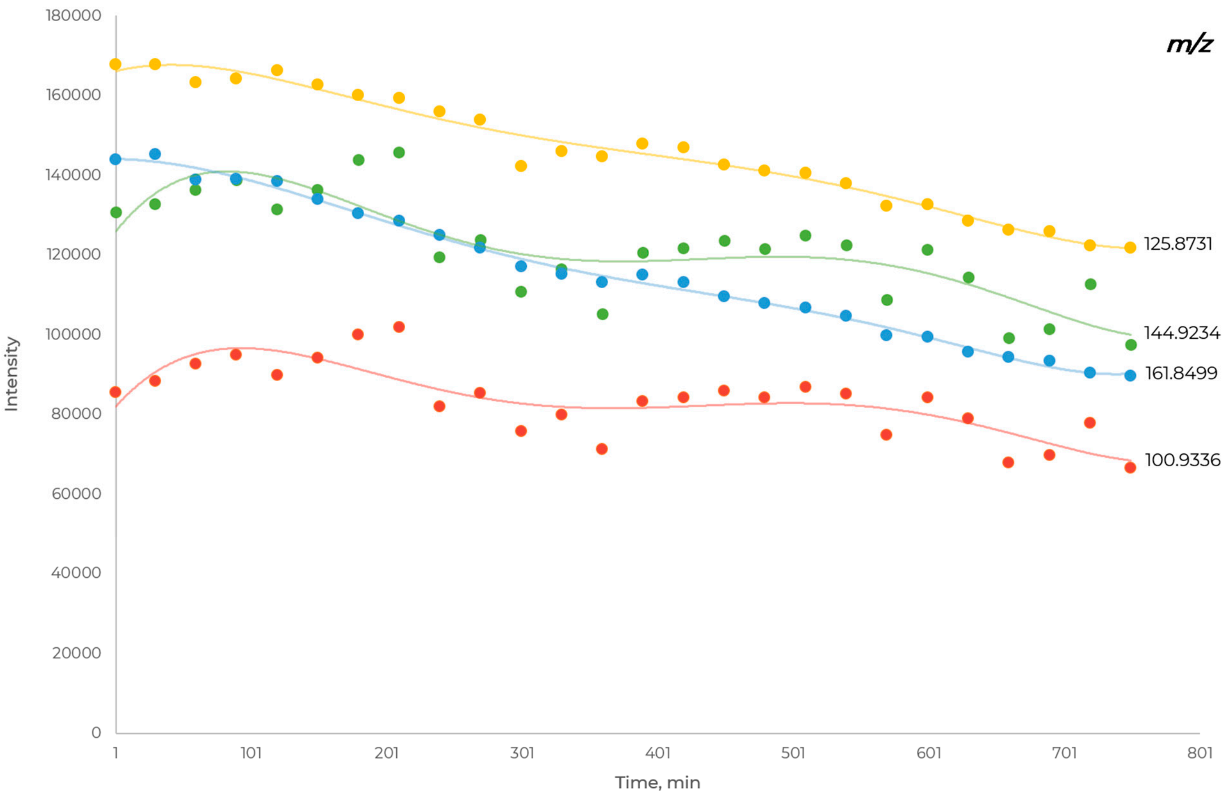

Figure S1. Examples of ion tracks with minimal dynamics. The dots in the graph represent the measured intensity values at specific time points for the corresponding *m*/*z* values. The solid curves represent fifth-order polynomial fits used solely as visual guides to facilitate recognition of the overall intensity shifts.

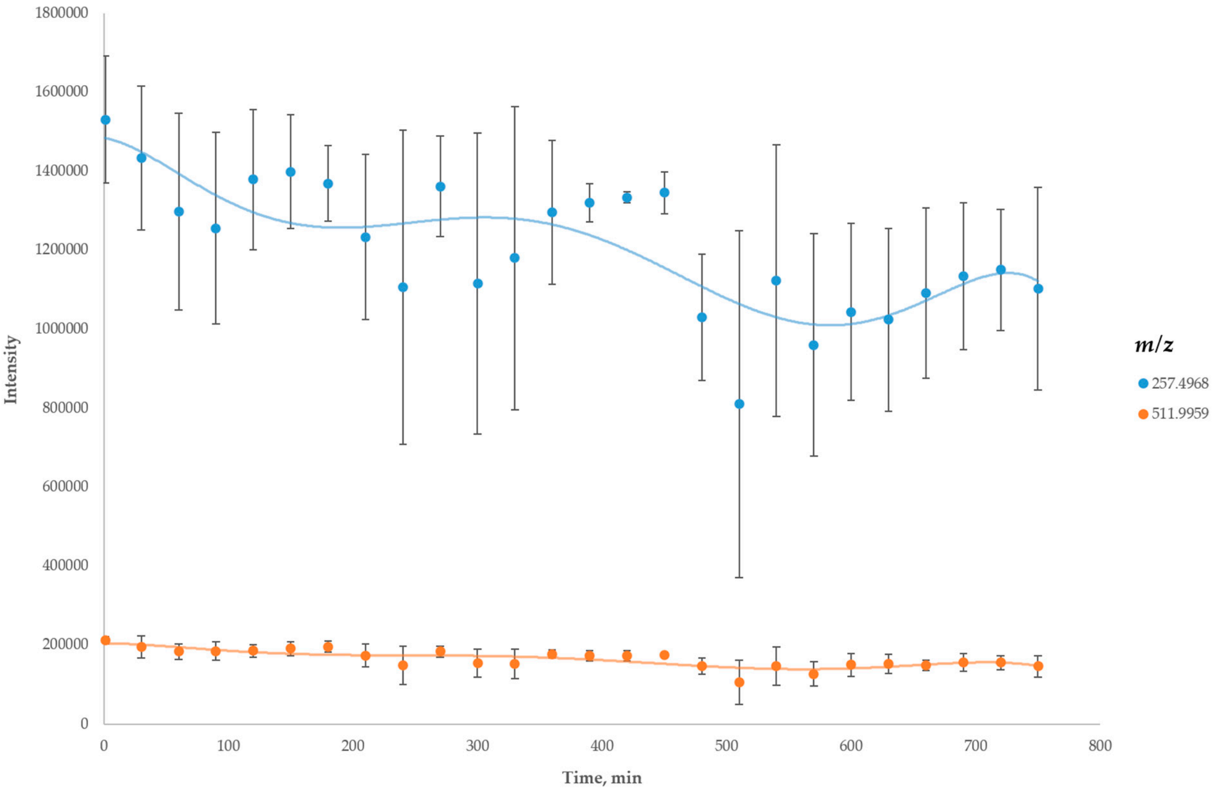

Figure S2. Ions that didn't show any sustainable pattern but passed the data funnel analysis. The dots in the graph represent the measured intensity values at specific time points for the corresponding *m/z* values. The solid curves represent fifth-order polynomial fits used solely as visual guides to facilitate recognition of the overall intensity shifts.
